# Rare complication: refractory hypertension and intermittent claudication caused by elephant trunk entrapped in a new entry after total arch replacement for type A aortic dissection

**DOI:** 10.1186/s44215-023-00050-5

**Published:** 2023-06-07

**Authors:** Takayuki Fujii, Noriyuki Abe, Takahiro Yamazato, Noriko Ohyama, Hiroshi Munakata

**Affiliations:** Department of Cardiovascular Surgery, Okinawa Nanbu Prefectural Medical Center and Children’s Center, 118-1 Arakawa, Haebaru-Cho, Shimajiri-Gun, Okinawa, Japan

**Keywords:** Elephant trunk, Thoracic endovascular aortic repair, Graft induced new entry

## Abstract

**Background:**

Total arch replacement using elephant trunk (ET) has been accepted as a standard technique for thoracic aortic dissection. However, there are few complications related to the ET, such as kinking of the ET, paraplegia, splitting of the anastomosis, and thromboembolic complications. We report a successful thoracic endovascular aortic repair (TEVAR) in a patient with ET entrapment in a new isolated dissecting aortic aneurysm.

**Case presentation:**

A 50-year-old woman who underwent total arch replacement (TAR) with the ET technique 6 years ago was admitted with refractory hypertension and heart failure. Magnetic resonance angiography revealed that the ET was entrapped in an isolated dissecting aortic aneurysm, which obstructed blood flow, thus causing ischemia. She underwent TEVAR to reset the entrapment of the ET. After TEVAR, ischemic symptoms were immediately relieved.

**Conclusion:**

We present a case of new entry after the repair of a type A acute aortic dissection using ET, which rapidly progressed to a distal arch dissecting aneurysm and dislocation of the ET.

**Supplementary Information:**

The online version contains supplementary material available at 10.1186/s44215-023-00050-5.

## Background

In 1983, Hans Borst described the elephant trunk (ET) technique for aneurysm of the thoracic aorta, which had the advantage of making the second operation much easier [1]. Some complications related to the ET technique have been reported, such as kinking of the ET, spinal cord ischemia, thromboembolic complications, entrapment of the trunk in the dissected aortic lumen, and splitting of the anastomosis due to enlargement of the false lumen. Here, we describe a successful treatment with thoracic endovascular aortic repair (TEVAR) for a patient with entrapment of the ET in the new isolated dissecting aortic aneurysm after total arch replacement for Stanford type A aortic dissection.

## Case presentation

**Fig. 1:**
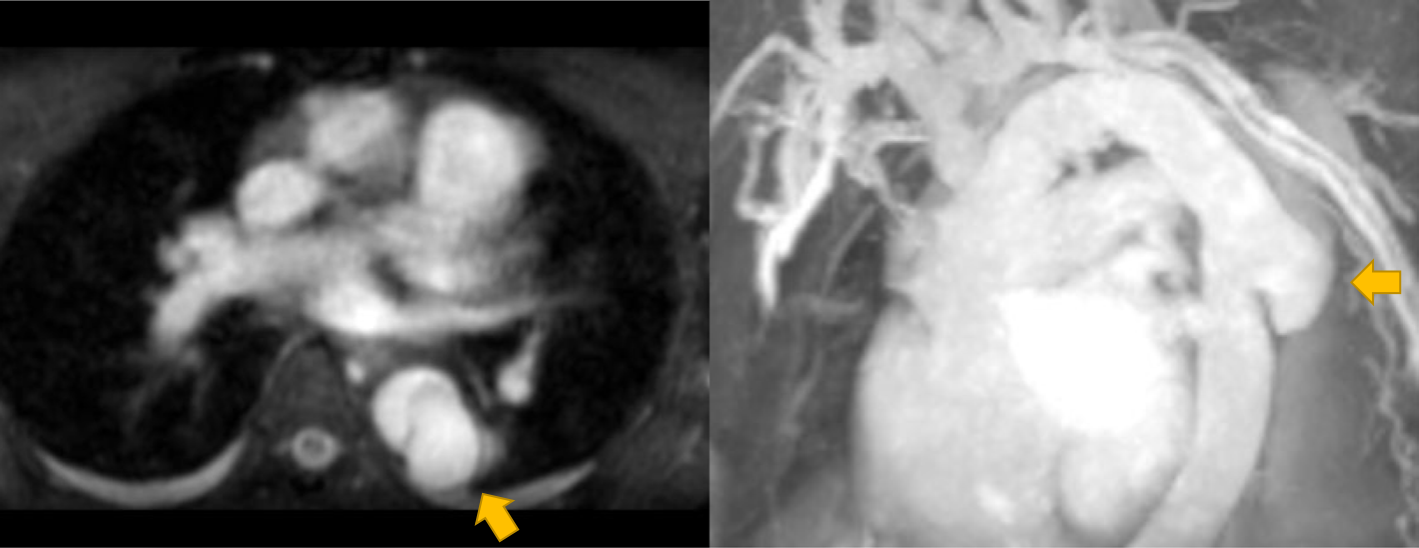
Preprocedural magnetic resonance angiography (MRA) revealed that the distal elephant trunk (ET) was entrapped in the isolated dissecting aortic aneurysm. An arrow designates the entrapped ET

A 50-year-old woman was admitted to another hospital because of hypertensive heart failure. Three months later, she was referred to our hospital for further evaluation and treatment of intermittent claudication, refractory hypertension, and acute kidney injury (Cre 2.25 mg/dl, eGFR 19.2 ml/min/1.73 m^2^). She underwent total arch replacement with the ET technique (Hemashield 4 branch aortic arch graft 24 mm, Getinge Maquet, Rastatt, Germany and Gelweave 22 mm, Terumo Corp, Tokyo, Japan) for acute type A aortic dissection 6 years ago. Her hypertension has worsened in 1 year, and she was administered a calcium channel blocker, beta-blocker, alpha-blocker, angiotensin receptor blocker (ARB), and diuretics for refractory hypertension. Plain computed tomography (CT) and magnetic resonance angiography (MRA) revealed that the ET was entrapped in an isolated dissecting aortic aneurysm (Fig. 1). Entrapment of the ET, which obstructed blood flow, was considered the cause of the ischemic symptoms.

**Fig. 2:**
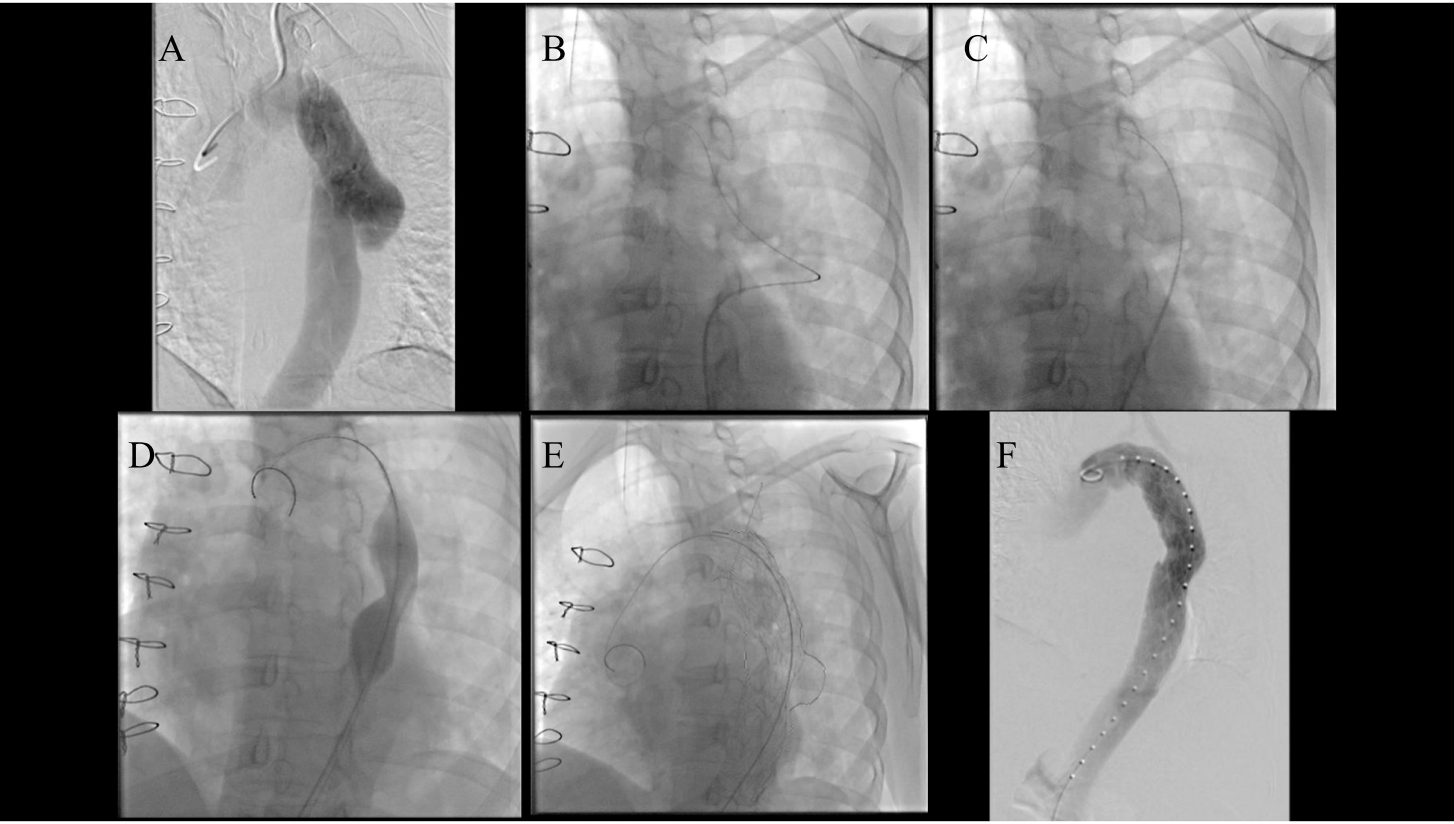
Intraoperative angiography of TEVAR to reset the entrapment of the ET. **A** Initial angiography showed that ET was entrapped in the isolated dissecting aortic aneurysm. **B** A guidewire was used to select the ET. **C** The guidewire was exchanged for a Lunderquist wire. **D** The balloon was dilated to reset the ET. **E** A Stent graft was deployed with postdilatation. **F** Completion angiography showed that entrapment of the ET was reset and excluded isolated dissecting aortic aneurysms

She underwent percutaneous TEVAR to reset the entrapment of the ET under general anesthesia. After insertion of an 8 Fr sheath, unfractionated heparin (4000 units) was injected into the vein. A 0.035 Radi-focus guidewire (Terumo Corp, Tokyo, Japan) and a 4.2 Fr selective catheter (Cobra-type) (Hanaco Medical, Saitama, Japan) were used for to select the ET. The guidewire was exchanged for a Lunderquist wire (Cook, IN, USA). After the MAXI-LD balloon 25 mm (Cordis, FL, USA) was dilated to reset the ET, CTAG TGM262615J (W.L. Gore, AZ, USA) was deployed with postdilatation. Finally, completion angiography showed that entrapped ET was reset and excluded isolated dissecting aortic aneurysms (Fig. 2). After TEVAR reset the entrapment of the ET, the intermittent claudication, refractory hypertension, and acute kidney injury were immediately relieved (Supplementary Fig. 1) The institutional IRB did not approve this study, because IRB approval is not needed for case reports in our institute. Consent for this report was provided by the patient.

## Discussion and conclusion

According to the operative report, the patient was 44-year-old woman who underwent TAR with ET for type A aortic dissection which extended descending aorta, and the primary tear was located in ascending aorta. The diameter of descending aorta was 24 mm, and Gelweave 22 mm was used as elephant trunk. There is controversy as to whether proximal aortic replacement (PAR) or TAR is the more optimal surgical approach for acute type A aortic dissection. Although TAR is a challenging procedure, TAR might reduce the risk of distal aneurysm formation and late complication rate. In the current meta-analysis, it was demonstrated that, although PAR might result in beneficial perioperative and early postoperative outcomes, TAR is associated with a significant survival benefit at the long-term follow-up [2]. Frozen elephant trunk technique (FET) has become a valid option to treat acute type A aortic dissection. The use of FET can help to expand and stabilize the true tear and cover eventual supplementary ones in the stented portion of the aortic arch or proximal descending aorta [3]. Therefore, we choose TAR with FET for acute type A aortic dissection. However, a tear-oriented strategy is indicated in older patients with limited dissections and those presenting in less stable clinical conditions. In this case, the reason why FET was not used is unknown, because the operator of the initial surgery no longer belongs in our institution.

**Fig. 3:**
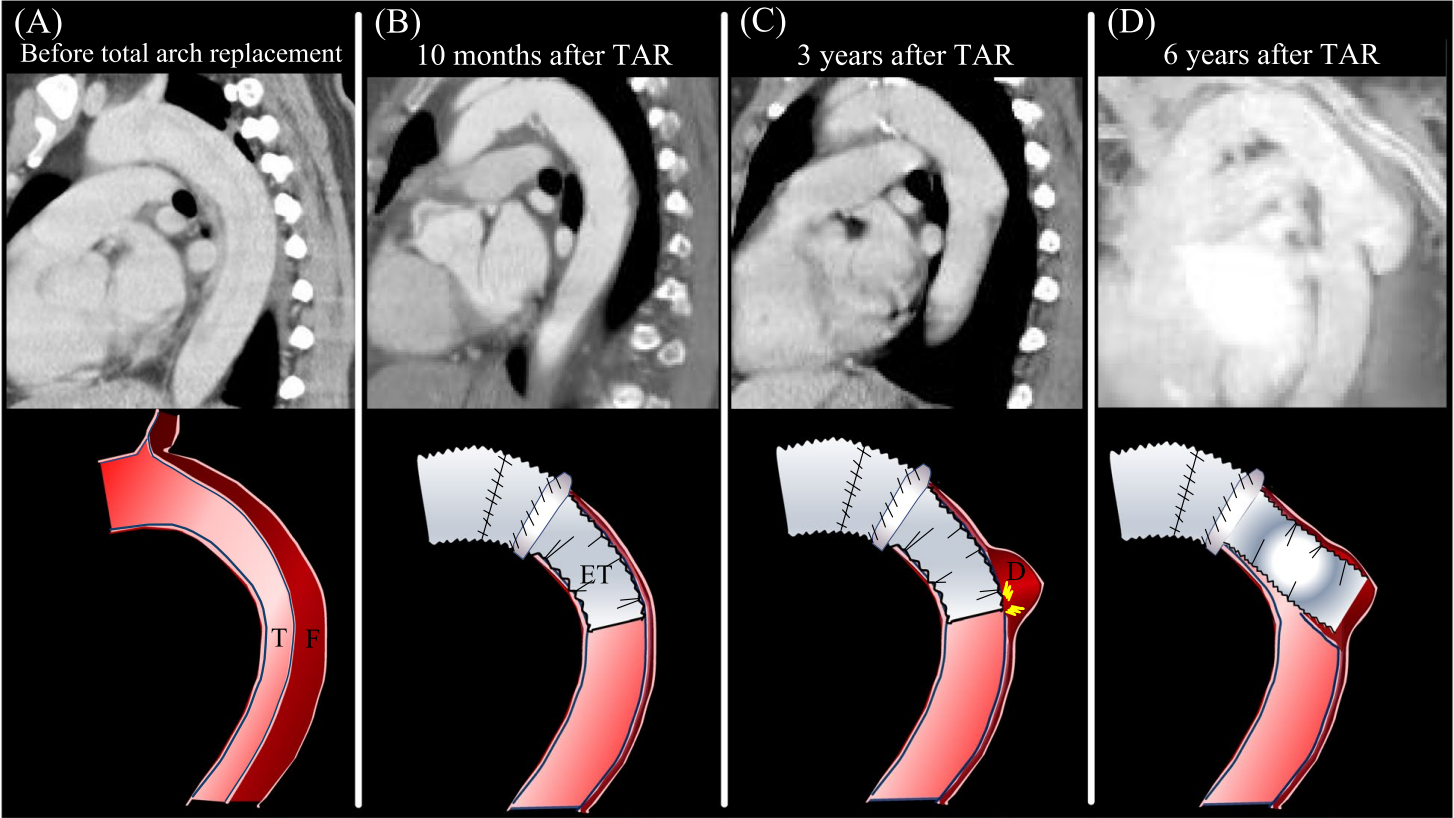
A time course of aortic configurations on enhanced computed tomography images and schemas. **A** Total arch replacement (TAR): persistent false lumen and forced true lumen. **B** Ten months after TAR with elephant trunk (ET): aortic remodeling was achieved, and the false lumen had almost disappeared. **C** Three years after TAR with ET: new entry occurred in the site of the distal ET. **D** Six years after TAR with ET: the distal ET was entrapped in the isolated dissecting aortic aneurysm

The ET technique is currently widely used. The original technique developed by Borst and colleagues was modified by Crawford and colleagues with placement of the inverted graft in the descending aorta and extension of the free portion into the descending aorta. In early experience, this treatment was applied only to patients with aneurysmal disease and was later extended to those with aortic dissection. The ET technique for repairing aortic dissection makes secure anastomosis with the distal friable tissue possible and prevents suture hole leaks [4, 5]. Reported complications related to the ET procedure are kinking obstruction, embolization, graft entrapment in the false lumen, and paraplegia [6, 7]. However, entrapment of the ET isolated new dissected aorta has not been described previously. We considered that a stentless graft, as an elephant trunk, may have induced new entry (GINE) because there was no re-entry near the ET before TAR on enhanced CT image. Moreover, hypertension was controlled well after initial surgery, the false lumen was very localized at the site of ET, and aortic remodeling was completely achieved once (Fig. 3) Although we suspected fragility of the aortic wall, such as Marfan syndrome, she had no family history of aortic dissection, and histopathology showed no cystic medial necrosis. We hypothesized that the tight edge made by folding the ET caused continuous stress to the aortic wall and made a new entry into the descending aorta (Supplementary Fig. 2). However, there is also a possibility that a new aortic dissection occurred accidentally at the site of ET. The cause of occurrence of the new entry could not be clear. The diameters of the descending aorta after remodeling and the ET were 21 mm and 22 mm, respectively. We estimated that the size of the ET was appropriate; therefore, this complication was unavoidable. To our knowledge, this is the first report of an entrapped ET in a new isolated dissected aorta. Although very rare complication, blood flow obstruction and lower body ischemia can occur by entrapment of the ET isolated new dissected aorta after surgery with stentless grafts as the ET, as demonstrated in our case report. To find this complication, we recommend careful attention to the occurrence of new entry in the edge of an ET during follow-up.

## Supplementary Information

**Additional file 1:** **Figure 1.** Post-thoracic endovascular aortic repair computed tomography.

**Additional file 2:** **Figure 2.** Preprocedural 3-dimensional computed tomography angiography. The red arrow designates folds of the elephant trunk.

## Data Availability

Not applicable.

## Abbreviations

ET: Elephant trunk
TEVAR: Thoracic endovascular aortic repair
GINE: Graft induced new entry
MRI: Magnetic resonance angiography
