# Supplementary figures and images for: Rare complication: refractory hypertension and intermittent claudication caused by elephant trunk entrapped in a new entry after total arch replacement for type A aortic dissection
Source: Gen Thorac Cardiovasc Surg Cases. 2023 Jun 7;2:40. doi: 10.1186/s44215-023-00050-5 (PMC11533691; doi:10.1186/s44215-023-00050-5)

## Slide 1
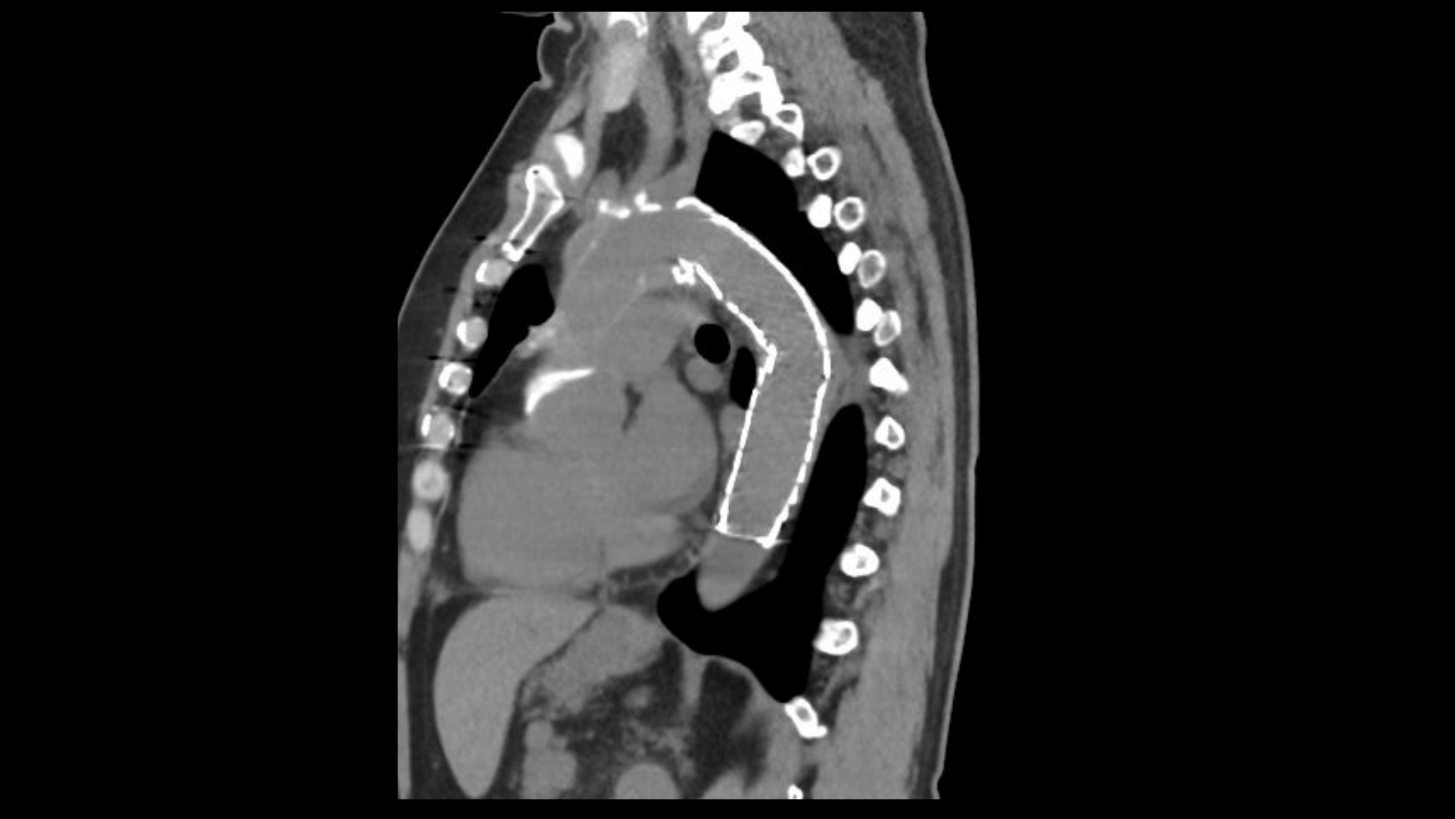

Supplement: Supplementary file 1 — Additional file 1: Figure 1. Post-thoracic endovascular aortic repair computed tomography. [file 44215_2023_50_MOESM1_ESM.pptx]

## Slide 1
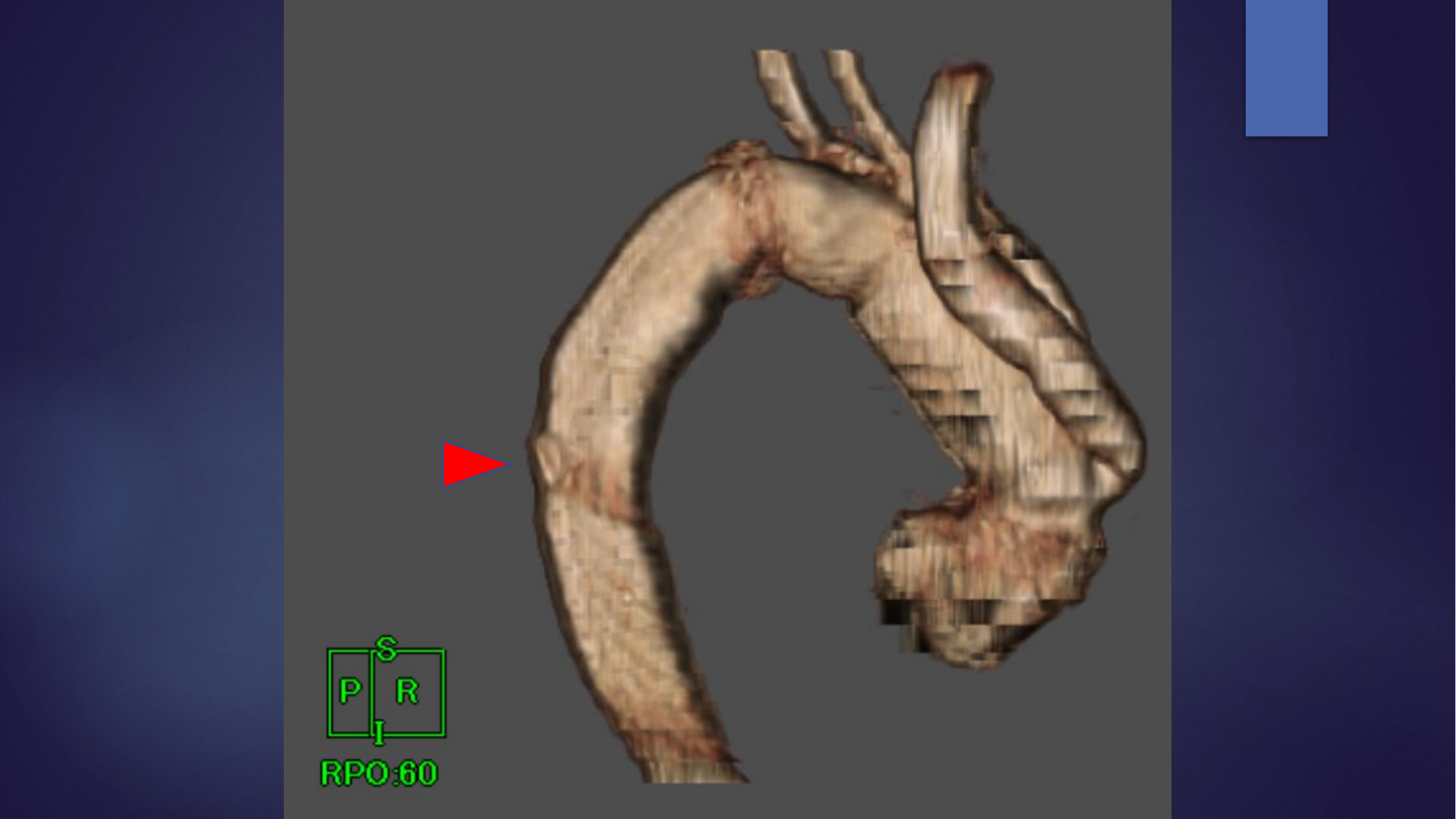

Supplement: Supplementary file 2 — Additional file 2: Figure 2. Preprocedural 3-dimensional computed tomography angiography. The red arrow designates folds of the elephant trunk. [file 44215_2023_50_MOESM2_ESM.pptx]
